# Application of cognitive behavioural therapy combined with aripiprazole in the treatment of schizophrenia: a randomised controlled trial

**DOI:** 10.1017/neu.2025.14

**Published:** 2025-03-27

**Authors:** Jun Yan, Kunjie Li, Qiang He, Jie Xiong

**Affiliations:** 1 Department of Psychiatry, The Second People’s Hospital of Guizhou Province, Guiyang Guizhou, China.

**Keywords:** schizophrenia, cognitive behavioural therapy, Aripiprazole, cognitive function, psychological state

## Abstract

This study focused on the effect of the cognitive behavioural therapy (CBT) combined with aripiprazole on cognitive functions and psychological state of schizophrenia patients. Seventy-eight schizophrenia patients were divided into two groups. One group received aripiprazole with conventional nursing treatment for 3 months (control group, *n* = 39), and the other received aripiprazole with CBT for 3 months (observation group, *n* = 39) (1 session per week, each session lasting 60 min. In the two groups before and after treatment, the severity of symptoms was evaluated using the Psychiatric Symptom Rating Scale (BPRS). Cognitive function was assessed with the Repeatable Battery for the Assessment of Neuropsychological Status (RBANS). The Positive and Negative Symptom Scale (PANSS) was utilised to evaluate mental status, while the Generalised Self-Efficacy Scale (GSES) measured psychological state. Additionally, the quality of life was assessed using the General Quality of Life Inventory-74 (GQOLI-74). In the final analysis, post-treatment efficacy and complications for the two groups were counted. Both groups showed significant improvements: BPRS and PANSS scores decreased, while RBANS, GSES, and GQOLI-74 scores increased. The observation group showed greater improvements than the control group. The total improvement rate was 89.74% (35/39) in the observation group, higher than the 71.79% (28/39) in the control group. The complication rate was 33.33% (13/39) in the observation group and 38.46% (15/39) in the control group. The treatment of CBT combined with aripiprazole for schizophrenia has a significantly positive effect on the cognitive functions and psychological state of patients.

## Graphical abstract

**Figure:**
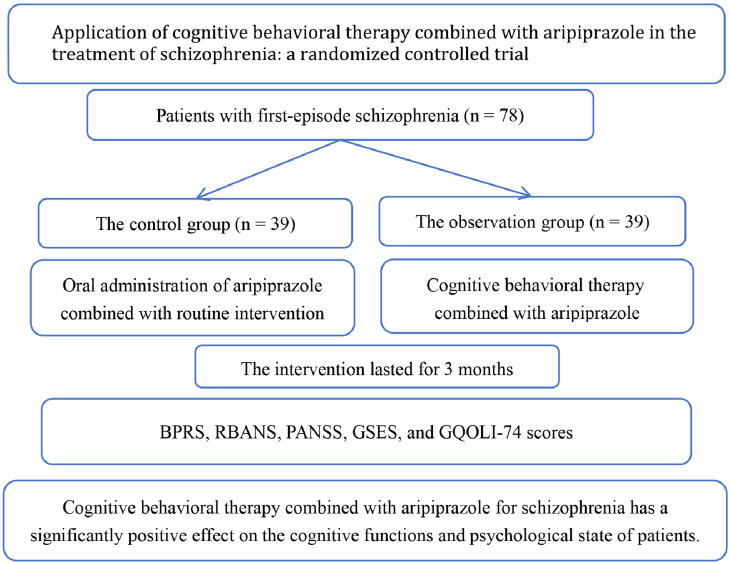

Significant outcomes

- Cognitive behavioural therapy (CBT) and aripiprazole alleviate the severity of symptoms in schizophrenia patients.
- CBT and aripiprazole improve cognitive function in schizophrenia patients.
- CBT and aripiprazole improve the mental state of schizophrenia patients.

Limitations

- It is worth noting that the study is based on limited clinical data, which is a major limitation.
- Larger-scale studies with longer follow-up periods are needed to confirm the findings and explore the long-term effects of cognitive behavioural therapy combined with aripiprazole in schizophrenic patients.
- Additional potential mechanisms for improved cognitive and psychological outcomes were not addressed in this study.

Highlights

- Cognitive behavioural therapy (CBT) and aripiprazole alleviate the severity of symptoms in schizophrenia patients.
- CBT and aripiprazole improve cognitive function in schizophrenia patients.
- CBT and aripiprazole improve the mental state of schizophrenia patients.
- CBT and aripiprazole improve the psychological state of schizophrenia patients.
- CBT and aripiprazole improve the quality of life of schizophrenia patients.

## Introduction

Schizophrenia is a genetically influenced, multifaceted syndrome characterised by diverse psychotic, negative, cognitive, mood, and motor symptoms. The condition follows a fluctuating course, with varying levels of recovery among affected individuals, most of whom experience substantial social and functional limitations (Tandon *et al*., 2024). The prevalence of schizophrenia-spectrum disorders with onset before the ages of 14, 18, and 25 is 3%, 12.3%, and 47.8%, respectively, peaking at around 20.5 years of age and with a median onset age of 25 years (Solmi *et al*., 2023). Schizophrenia typically emerges in young adults, characterised by disturbances in perception, cognition, emotions, and behaviour, including positive symptoms, negative symptoms, and cognitive impairment (Xu & Zhang, 2023). These impairments are a central aspect of schizophrenia, contributing substantially to functional disability and are resistant to current treatments (McCutcheon *et al*., 2023). Consequently, understanding and addressing cognitive dysfunction in schizophrenia remains a significant challenge (Lu *et al*., 2023). Additionally, schizophrenia is characterised by positive symptoms like delusions, hallucinations, and disorganised thought processes, as well as negative symptoms such as reduced speech, social isolation, and decreased emotional expression. The broad range of cognitive and neurophysiological impairments associated with schizophrenia has a profound impact on quality of life and social functioning (Singh *et al*., 2024).

Antipsychotic medications are among the most effective treatments for schizophrenia spectrum disorders (Li *et al*., 2024). Aripiprazole, a third-generation antipsychotic approved by the US Food and Drug Administration, is used for treating schizophrenia and is available in both oral and long-acting injectable formulations (Preda & Shapiro, 2020). Aripiprazole is a third-generation antipsychotic that is typically well-tolerated, with a low incidence of motor side effects and metabolic issues commonly seen with other antipsychotic medications (Preda & Shapiro, 2020). Aripiprazole, a ‘dopamine system stabiliser’, partially agonises dopamine D2 and serotonin-5-HT_1A_ receptors while antagonising serotonin-5-HT_2A_ receptors. It demonstrates strong clinical efficacy with a favourable safety and tolerability profile in schizophrenia patients (Qian *et al*., 2021). The choice of aripiprazole was driven by its clinical relevance, efficacy, and favourable safety profile. Aripiprazole is commonly prescribed either alone or in combination with other second-generation antipsychotics. However, this can increase the risk of clinically significant drug interactions (Jiang *et al*., 2021). Despite its effectiveness, over 80% of patients taking oral antipsychotics experience relapses, often due to non-adherence, which highlights the need for additional treatment strategies beyond medication alone (Lhaglham *et al*., 2024). Cognitive behavioural therapy (CBT) is a well-established treatment for various psychological disorders, including anxiety, depression, and schizophrenia (Hassan Kariri & Almubaddel, 2024). Extensive research has shown that CBT is effective in numerous outcome studies for conditions such as depression, anxiety disorders, eating disorders, substance abuse, and personality disorders. It has also been proven effective as an adjunct to medication for serious mental illnesses like bipolar disorder and schizophrenia (Chand *et al*., 2024). Anthony P Morrison *et al*. have supported that CBT is well-received, leading to modest but meaningful improvements in psychiatric symptoms at the end of treatment (9 months) and sustained progress in self-rated recovery (21 months), with minimal evidence of adverse effects (Morrison *et al*., 2018). Meanwhile, CBT has shown promise as an effective adjunct treatment, improving social adaptation, quality of life, and reducing symptoms of mental illness and associated distress in schizophrenia (Abdel-Baki & Nicole, 2001).

At present, there are few studies to explore the effect of CBT combined with aripiprazole on cognitive function and psychological state for schizophrenia patients. While aripiprazole addresses the pharmacological aspects of schizophrenia, CBT targets cognitive and negative symptoms, improving coping strategies and social functioning. This combination offers potential synergistic effects, enhancing treatment adherence and long-term outcomes. Based on this, the present paper aims to explore the effect of CBT combined with aripiprazole on cognitive function and psychological state of schizophrenia patients by the random number table method and scale evaluation.

## Materials and methods

### Ethical statement

This study was ratified by the ethics committee of The Second People’s Hospital of Guizhou Province (approval number: 2020018). Informed consent was signed by the families of all participating patients. This study was conducted in Guizhou, China.

### General demographic characteristics

From September 2020 to October 2021, 78 schizophrenia patients admitted to The Second People’s Hospital of Guizhou Province were divided by the random number table method into a control group and an observation group with 39 cases each. The discrepancies of general demographic characteristics between the two groups were not significantly great (*p>*0.05), which was comparable (Table 1).

**Table 1.** Comparison of general demographic characteristics between the two groups

Indicator	Observation group (*n* = 39)	Control group (*n* = 39)	*Z/χ* ^2^	*p*
Age (years)	22.00 (20.00, 25.00)	23.00 (19.00, 26.00)	−0.080	0.936
Gender (*n*)			0.212	0.645
Male	22 (56.41)	24 (61.54)	–	–
Female	17 (43.59)	15 (38.46)	–	–
Years of education (*n*)			1.471	0.479
≤9 years	16 (41.03)	19 (48.72)	–	–
10 ∼ 12 years	20 (51.28)	15 (38.46)	–	–
≥13 years	3 (7.69)	5 (12.82)	–	–
Marital status			0.075	0.784
Married	9 (23.08)	8 (20.51)	–	–
Unmarried/divorced/widowed	30 (76.92)	31 (79.49)	–	–
Residence			0.241	0.624
Rural areas	13 (33.33)	11 (28.21)	–	–
Urban areas	26 (66.67)	28 (71.79)	–	–
Professional character			2.151	0.828
Intellectual workers	12 (30.77)	9 (23.08)	–	–
Manual workers	8 (20.51)	10 (25.64)	–	–
Worker in service industries	6 (15.38)	5 (12.82)	–	–
Freelancers	4 (10.26)	2 (5.13)	–	–
The unemployed	6 (15.38)	9 (23.08)	–	–
Students	3 (7.69)	4 (10.26)	–	–
Smoking history			0.482	0.488
Yes	25 (64.10)	22 (56.41)		
No	14 (35.90)	17 (43.59)		
Drinking history			0.466	0.495
Yes	20 (51.28)	23 (58.97)		
No	19 (48.72)	16 (41.03)		

### Inclusion criteria

① Well-defined diagnostic criteria for schizophrenia (Dilling & Dittmann, 1990). ② Age ranging from 18 to 40 years old. ③ First episode, confirmed no issues with antipsychotic drug tolerance. ④ Stable basic vital signs of the patient, with no impediments in verbal expression and basic limb activities. ⑤ Assessed to have no severe suicidal ideation, aggressive behaviour, or acute mental symptoms. ⑥ The patient himself/herself or the legal guardian agreed to participate in the study and able to cooperate with the treatment and assessment process of the study. ⑦ Approved by hospital ethics.

### Exclusion criteria

① Concurrent with other mental disorders, such as bipolar disorder, depression, anxiety disorder, mental retardation, drug dependence, etc. ② Complicated with severe neurological diseases, such as epilepsy, cerebral infarction, Parkinson’s disease, Alzheimer’s disease, and other neurological diseases that might significantly affect cognitive function. ③ Pregnancy or lactation period. ④ Poor adherence to medical advice or recent receipt of other psychological treatments. ⑤ Assessed to have severe cognitive deficits or inability to understand the treatment content and scale tests normally. ⑥ Presence of alcoholism, drug abuse, or psychotropic drug misuse. ⑦ Concurrent participation in other intervention studies or withdrawal due to various factors.

### Methods

(1) Control group: Oral administration of aripiprazole (Shanghai Shangyao Chinese-Western Pharmaceutical Co., Ltd.; No. H20041506; Specifications: 5 mg). The initial dose was taken once daily, 5–10 mg each time. The dosage could be adjusted according to the patient’s condition, and the increased dosage should not exceed 30 mg/d. This treatment lasted for 3 months. Conventional interventions during the treatment period included enhancing communication with the patients after admission, providing understanding, support, and attention with a warm and empathetic attitude; increasing awareness of schizophrenia and helping patients normalise their individual situations, ensuring they fully understand and correctly recognise the psychotic symptoms they are experiencing, thereby reducing the impact on related functions and psychological well-being; encouraging family members to provide more understanding, care, and companionship to the patients; and assisting in improving the intimacy between the patients and their family members to boost their confidence in life.

(2) Observation group: Treatment of CBT combined with aripiprazole. CBT: A CBT team was established, consisting of one clinical doctor, one psychological counsellor, and three specialised nurses. All team members possessed relevant qualifications in psychological therapy, had received professional training, and passed the assessments. The team participated in weekly case supervision sessions. The team members provided CBT to the patients. ① Symptom education: Patients were guided to understand the characteristics, symptom types of the disease, and its impact on life. For example, the biological and psychological causes of schizophrenia were explained to the patients; patients were made aware that symptoms such as hallucinations and delusions were pathological rather than realistic events; the patient was empowered to better understand and feel less ashamed about the disease. ② Early symptom identification: Patients and their family members were guided to identify early signs of disease recurrence. For example, by recalling the medical history, patients were guided to discover early symptoms (such as depression, sleep disorders, or decreased attention). ③ Medication compliance intervention: Patients were educated to know better about the importance of medication and act in accordance with their doctors. For example, patients were told about the possible positive and side effects of drugs to eliminate misunderstandings or fears; patient’s medication was recorded, difficulties in the treatment were discussed, and problems were solved with joint efforts; a reasonable medication plan was formulated and reminder mechanisms (such as pill boxes or mobile phone alarms) were set up. ④ Intervention for hallucinations: Patients were helped to re-evaluate the source and meaning of the heard voices or other hallucinations. For example, patients were taught to use the ‘voice discrimination’ technique (distinguishing hallucinations from real sounds); patients were guided to record the frequency, time, and triggers of hallucinations so as to identify their regularity; patients were guided to view hallucinations as ‘bystanders’ to reduce emotional responses. ⑤ Intervention for delusions: Patients were helped to challenge and correct the unreasonable belief system (such as persecution delusions and grandiosity delusions). For example, patients were guided to discuss the specific content of their delusions and explore whether these beliefs were logical through questioning; patients were asked to list the evidence supporting and refuting the delusions to help them understand their cognitive biases through the evidence comparison method; patients were encouraged to use alternative explanations (such as thinking of ‘maybe a coincidence’ instead of ‘being monitored’). ⑥ Emotional regulation: Patients were helped to regulate adverse emotional states. For example, relaxation training, such as deep breathing, muscle relaxation, and mindfulness meditation, was conducted; skills of identifying and expressing emotions to avoid excessive suppression or intensification of emotions were taught; patients’ overly negative interpretations of setbacks or failures were corrected through cognitive restructuring. ⑦ Stress response: Patients were helped to improve their ability to cope with stressors and difficulties in daily life. For example, they were taught to develop problem-solving skills (e.g. breaking down and prioritising problems); coping strategies and emergency plans were made to help patients remain calm in high-pressure environments; training in social skills (e.g. interacting with others and asking for help) was provided. ⑧ Cognitive reconstruction: Patients were helped to change negative thinking patterns and enhance self-efficacy, such as identifying their negative automated thoughts (such as ‘I have no value’); patients were guided to correct these cognitive errors by showing the evidence about what proved oneself worthless; patients were assisted to replace negative thinking with positive and realistic beliefs. ⑨ Behavioural activation: Patients were guided to improve their passive or isolated living conditions and become more enthusiastic about life. For example, patients were encouraged to participate in meaningful activities (such as practising hobbies and volunteer services); specific and achievable small goals were set to increase patients’ sense of accomplishment; a daily schedule was established to increase the regularity and efficiency of activities. ❿ Social functional training: The patient’s social interaction ability and social adaptation ability were improved by such training as conducting scenario simulation training and teaching basic communication skills (such as how to start and end conversations); patients were guided to practice skills in handling interpersonal conflicts in a safe environment; simulation training of work scenarios was offered to help patients gradually adapt to employment. ⑪ Family interventions: Family members’ ability was enhanced to support patients and reduce the risk of relapse, such as teaching family members the skills of effective communication and emotional expression, and reducing conflicts; disease education was supplied to help family members understand patients’ symptoms and behaviours; a family support plan was put forward to assist patients in establishing a stable, safe living environment. ⑫ Relapse prevention and long-term planning: The risk of relapse was reduced. The patient’s long-term recovery was supported by teaching both the patient and his or her family to develop a relapse response plan (such as how to deal with early signs); regular return visits were paid to evaluate the patient’s psychological state and social function; patients were encouraged to participate in community rehabilitation programmes or support groups to maintain therapeutic outcomes. Each treatment session lasted 60 min, with a frequency of 1 session per week. This treatment with CBT lasted for 3 months. The usage and dosage of aripiprazole were the same as above. During the intervention period, the team members remained unchanged, and all patients successfully completed the treatment with no dropouts.

### Observation indicators

1. Brief Psychiatric Rating Scale (BPRS): Before and after treatment in the two groups, the BPRS was used to assess the symptom severity of patients. This scale generally consisted of five symptom factors, namely, anxiety and depression (4 questions), lack of vigour (4 questions), thought disorder (4 questions), activation (3 questions), and hostility and suspiciousness (3 questions), with a score of 1–7 points for each query, and a total of 18–126 points. It stipulated that the higher total score means being associated with the worse the symptom (Morlan & Tan, 1998).
2. Repeatable Battery for the Assessment of Neuropsychological Status (RBANS): Before and after treatment in the two groups, the RBANS was adopted to evaluate the cognitive function of patients. This scale generally included five cognitive dimensions, namely immediate memory, Visual-spatial competence, language, attention, and delayed memory. The scores of each dimension were converted from the original scores to the standardised scores, with a mean of 100 points and a standard deviation of 15 points. The higher the total score, the better the cognitive function (Novitski *et al*., 2012).
3. Positive and Negative Syndrome Scale (PANSS): Before and after treatment in the two groups, PANSS was utilised to assess the mental state of patients. This scale generally included three sub-scales, namely, negative symptoms (7 questions), positive symptoms (7 questions), and general pathological symptoms (16 questions). Each question was scored 1–7 points, totalling 30–210 points. The higher the total score, the worse the mental state (Kay *et al*., 1987).
4. Generalised Self-Efficacy Scale (GSES): Before and after treatment in both groups, the GSES was used to assess the psychological state of patients. This scale included 10 questions, each with a score of 1–4 points and a total score of 10–40 points. The higher the total score, the better the psychological state (Luszczynska *et al*., 2005).
5. Generic Quality of Life Inventory 74 (GQOLI-74): Before and after treatment in the two groups, the GQOLI-74 was implemented to assess the quality of life of patients. This scale generally included four dimensions: material life, physical function, psychological function, and social relationship. The total score of each dimension was 0 ∼ 100 points. The higher the total score, the better the quality of life (Hou *et al*., 2021).
6. Efficacy: After treatment, the clinical efficacy of the two groups of patients was evaluated by the reduction rate of score reduction of the PANSS (Kay *et al*., 1987). A reduction rate of ≥50% denoted significant improvement, and a reduction rate of 25∼49% indicated partial improvement, while a reduction rate <25% suggested no improvement. The reduction rate was calculated as (total score before treatment – total score after treatment)/total score before treatment × 100%, and the overall improvement rate was determined as (number of cases showing significant improvement + number of cases showing partial improvement)/total number of cases × 100%.
7. Complications: Statistical analysis of complications was made during treatment in the two groups, including those common complications related to the central nervous system, gastrointestinal tract, endocrine and metabolic systems, extrapyramidal symptoms, and cardiovascular system.

### Statistical methods

Statistical analysis was carried out by SPSS 26.0 (Loffing, 2022). Qualitative data were described by [*n* (%)] for *χ* ^2^ tests. Normally distributed quantitative data were described by 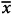 ± *s* for independent sample or paired *t* tests, and skewed quantitative data were described by *M*(*p25*, *p75*) for Mann*–*Whitney *U* tests. When a difference equalling *P* < 0.05 occurred, it was considered statistically significant.

## Results

### Symptom severity

Before treatment, the scores for each aspect of the BPRS showed no notable variance between the two groups (*p*>0.05). After treatment, there was a reduction in the scores for each aspect of the BPRS in both groups, with the observation group’s scores falling below those in the control group (*p*<0.05; Table 2).

**Table 2.** Comparison of symptom severity between the two groups (points)

Time	Observation group (*n* = 39)	Control group (*n* = 39)	*Z*	*p*
Before treatment				
Anxiety	15.00 (12.00, 17.00)	15.00 (12.00, 17.00)	−0.407	0.684
Inactivity	13.00 (11.00, 15.00)	13.00 (10.00, 14.00)	−0.689	0.491
Thought disorder	14.00 (12.00, 15.00)	14.00 (12.00, 15.00)	−0.736	0.461
Activation	12.00 (10.00, 13.00)	12.00 (9.00, 13.00)	−0.121	0.904
Hostility and suspiciousness	13.00 (12.00, 16.00)	14.00 (12.00, 16.00)	−0.081	0.936
After treatment				
Anxiety	9.00 (7.00, 10.00)*	11.00 (9.00, 12.00)*	−2.851	0.004
Inactivity	8.00 (6.00, 9.00)*	9.00 (8.00, 10.00)*	−3.357	0.001
Thought disorder	10.00 (8.00, 11.00)*	12.00 (10.00, 13.00)*	−2.532	0.011
Activation	9.00 (6.00, 10.00)*	11.00 (8.00, 12.00)*	−2.858	0.004
Hostility and suspiciousness	10.00 (8.00, 10.00)*	11.00 (9.00, 13.00)*	−3.054	0.002

*P<0.05 versus the same group before treatment.

### Cognitive function

Before treatment, the RBANS scores showed no notable variance between the two groups (*p*>0.05). After treatment, there was a rise in the RBANS scores across both groups, with the observation group outperforming the control group (*p*<0.05; Table 3).

**Table 3.** Comparison of cognitive function between the two groups (points)

Time	Observation group (*n* = 39)	Control group (*n* = 39)	*Z*	*p*
Before treatment				
Immediate memory	64.00 (48.00, 70.00)	63.00 (49.00, 69.00)	−0.115	0.908
Visual-spatial competence	83.00 (67.00, 89.00)	78.00 (68.00, 87.00)	−0.300	0.764
Language	86.00 (81.00, 92.00)	88.00 (83.00, 93.00)	−0.580	0.562
Attention	81.00 (65.00, 87.00)	79.00 (64.00, 85.00)	−0.440	0.660
Delayed memory	70.00 (54.00, 76.00)	64.00 (54.00, 72.00)	−0.685	0.493
After treatment				
Immediate memory	74.00 (63.00, 80.00)*	69.00 (55.00, 75.00)*	−2.270	0.023
Visual-spatial competence	92.00 (81.00, 96.00)*	88.00 (74.00, 93.00)*	−2.201	0.028
Language	95.00 (93.00, 98.00)*	93.00 (87.00, 96.00)*	−2.756	0.006
Attention	89.00 (81.00, 95.00)*	83.00 (73.00, 89.00)*	−2.421	0.015
Delayed memory	81.00 (67.00, 86.00)*	73.00 (61.00, 79.00)*	−2.020	0.043

*P<0.05 versus the same group before treatment.

### Mental state

Before treatment, the PANSS scores showed no notable variance between the two groups (*p*>0.05). After treatment, there was a reduction in the PANSS scores across both groups, with the observation group’s scores falling below those in the control group (*p*<0.05; Table 4).

**Table 4.** Comparison of mental state between the two groups (points)

Time	Observation group (*n* = 39)	Control group (*n* = 39)	*Z*	*p*
Before treatment				
Negative symptoms	25.00 (21.00, 26.00)	24.00 (20.00, 26.00)	−0.577	0.564
Positive symptom	29.00 (24.00, 30.00)	28.00 (24.00, 30.00)	−0.582	0.561
General pathological symptoms	38.00 (34.00, 41.00)	38.00 (34.00, 40.00)	−0.827	0.408
After treatment				
Negative symptoms	17.00 (14.00, 17.00)*	19.00 (17.00, 20.00)*	−3.991	<0.001
Positive symptom	18.00 (14.00, 20.00)*	19.00 (17.00, 22.00)*	−2.285	0.022
General pathological symptoms	26.00 (22.00, 28.00)*	27.00 (25.00, 29.00)*	−2.093	0.036

*P<0.05 versus the same group before treatment.

### Psychological state

Before treatment, the GSES scores of both groups showed no notable variance (*p*>0.05). After treatment, the GSES scores rose in both groups, with the observation group’s scores surpassing those of the control group (*p*<0.05; Table 5).

**Table 5.** Comparison of psychological state between the two groups (points)

Time	Observation group (*n* = 39)	Control group (*n* = 39)	*Z*	*p*
Before treatment	27.00 (22.00, 29.00)	25.00 (23.00, 29.00)	−0.065	0.948
After treatment	34.00 (31.00, 37.00)*	29.00 (27.00, 33.00)^a*^	−3.951	<0.001

*P<0.05 versus the same group before treatment.

### Quality of life

Before treatment, the scores for each aspect of the GQOLI-74 showed no notable variance between the two groups (*p*>0.05). After treatment, there was a rise in the GQOLI-74 scores across both groups, with the observation group outperforming the control group (*p*<0.05; Table 6).

**Table 6.** Comparison of quality of life between the two groups (points)

Time	Observation group (*n* = 39)	Control group (*n* = 39)	*Z*	*p*
Before treatment				
Material life	46.00 (41.00, 49.00)	47.00 (42.00, 49.00)	−0.145	0.884
Physical function	47.00 (43.00, 49.00)	49.00 (44.00, 50.00)	−1.128	0.259
Psychological function	39.00 (35.00, 41.00)	41.00 (36.00, 42.00)	−1.113	0.266
Social relationship	41.00 (37.00, 43.00)	43.00 (38.00, 44.00)	−1.053	0.292
After treatment				
Material life	50.00 (47.00, 52.00)*	49.00 (44.00, 51.00)*	−2.070	0.038
Physical function	55.00 (50.00, 57.00)*	51.00 (47.00, 53.00)*	−2.670	0.008
Psychological function	52.00 (47.00, 55.00)*	48.00 (43.00, 50.00)*	−2.948	0.003
Social relationship	52.00 (46.00, 54.00)*	47.00 (42.00,49.00)*	−3.114	0.002

*P<0.05 versus the same group before treatment.

### Efficacy

After treatment, the observation group’s overall enhancement rate stood at 89.74% (35/39), surpassing the control group’s 71.79% (28/39) rate (*p*<0.05; Table 7).

**Table 7.** Comparison of efficacy between the two groups [n (%)]

Improvement	Observation group (*n* = 39)	Control group (*n* = 39)	*χ* ^2^	*p*
Significant improvement	20 (51.28)	12 (30.77)	–	–
Partial improvement	15 (38.46)	16 (41.03)	–	–
No improvement	4 (10.26)	11 (28.21)	–	–
Total improvement	35 (89.74)	28 (71.79)	4.044	0.044

### Complications

Throughout the course of the treatment, complication rates were 33.33% (13 out of 39) in the observation group and 38.46% (15 out of 39) in the control group (*p>*0.05; Table 8).

**Table 8.** Comparison of complications between the two groups [*n* (%)]

Complications	Observation group (*n* = 39)	Control group (*n* = 39)	*χ* ^2^	*p*
Insomnia	3 (7.69)	5 (12.82)	–	–
Dizziness and headache	2 (5.13)	3 (7.69)	–	–
Extrapyramidal symptoms	2 (5.13)	3 (7.69)	–	–
Nausea and vomiting	3 (7.69)	2 (5.13)	–	–
Constipation	2 (5.13)	2 (5.13)	–	–
Akathisia	2 (5.13)	3 (7.69)	–	–
Weight gain	1 (2.57)	0 (0.00)	–	–
Total	15 (38.46)	18 (46.15)	0.473	0.492

## Discussion

Individuals diagnosed with schizophrenia experience significant impairments in overall cognitive performance, with average scores typically falling two standard deviations below those of healthy controls (Keefe *et al*., 2011). Despite consistent scientific evidence linking schizophrenia to histories of traumatic life events and adversities, psychological therapies are often not offered to these patients (Magliano *et al*., 2016). Consequently, this study was aimed at analysing the effect of CBT combined with aripiprazole on cognitive function and psychological state of schizophrenia patients.

The results revealed a significant reduction in symptom severity, as measured by the BPRS, in both the control and observation groups after treatment. However, the observation group, which received CBT in addition to aripiprazole, showed a more substantial decrease in BPRS scores compared to the control group. This finding suggests that CBT may augment the efficacy of aripiprazole in alleviating the symptoms of schizophrenia. A previous study has shown that CBT can reduce relapse and hospitalization rates and increase well-being in individuals diagnosed with schizophrenia (Can & Budak, 2024). In terms of cognitive function, assessed using the RBANS, both groups exhibited improvements post-treatment. Notably, the observation group exhibited significantly higher RBANS scores than the control group, indicating better cognitive function. This outcome underscores the potential of CBT to enhance cognitive recovery in schizophrenic patients when used adjunctively with aripiprazole. Research has demonstrated that CBT is effective in treating various psychiatric disorders, including depression, anxiety, Post-Traumatic Stress Disorder (PTSD), and borderline personality disorder, and is frequently utilised in conjunction with medication (Saxena & Sahai, 2024).

Mental state, evaluated by the PANSS, also improved in both groups after treatment. However, the observation group demonstrated a more pronounced reduction in PANSS scores, indicating a greater improvement in mental status. This finding aligns with previous research indicating the beneficial effects of CBT on mental health outcomes in schizophrenic patients (Xu & Zhang, 2023). Furthermore, our study found improvements in psychological state and quality of life, as measured by the GSES and the GQOLI-74, respectively. Both groups showed increases in GSES and GQOLI-74 scores after treatment, with the observation group scoring significantly higher than the control group. This outcome is consistent with previous research showing that specific psychological treatments, such as CBT and Interpersonal Therapy, are effective and linked to lasting benefits following treatment (Grilo & Juarascio, 2023). Additionally, CBT, which is well-established as a psychological treatment for chronic pain, involves teaching individuals to challenge and restructure their maladaptive thinking patterns, enhance self-efficacy and coping strategies, and increase participation in life activities (Mikocka-Walus *et al*., 2021). We propose that there is a bidirectional relationship between cognitive skills and self-efficacy, where both constructs influence each other. On one hand, better cognitive skills, as measured by the RBANS, may enhance an individual’s ability to cope with challenges, which in turn can improve self-efficacy (as assessed by the GSES). On the other hand, increased self-efficacy can lead to more positive attitudes and greater motivation to engage in cognitive tasks, potentially leading to improvements in cognitive functioning over time. Therefore, we hypothesise that these constructs interact and mutually reinforce each other, contributing to overall recovery and improved quality of life.

Regarding efficacy, the observation group showed a higher overall improvement rate compared to the control group, based on the reduction rate of PANSS scores. This finding further supports the superiority of the combined treatment approach in managing schizophrenia and aligns with previous research proposing CBT as a beneficial adjunctive therapy to medication for individuals with schizophrenia (Guaiana *et al*., 2022). In terms of complications, the rates were comparable between the two groups, indicating that the addition of CBT to aripiprazole did not increase the risk of adverse events. This is an important consideration for clinicians when recommending treatment options for schizophrenic patients.

A review has found that combining individual counselling with CBT and motivational interviewing techniques effectively improved and sustained medication adherence over time (Inwanna *et al*., 2022). In addition, CBT has been found to improve medication adherence and reduce aggression in individuals with schizophrenia. It is recommended that psychiatric nurses incorporate CBT-based psychoeducation into their practices to enhance medication adherence and decrease aggression (Can & Budak, 2024). These psychosocial pathways could potentially enhance the effectiveness of aripiprazole by addressing both the cognitive and emotional challenges associated with schizophrenia.

In summary, this study contributes to the existing literature by providing evidence for the efficacy of CBT combined with aripiprazole in improving cognitive function and psychological state in schizophrenic patients. It lays a foundation to explore the relation between aripiprazole only and CBT combined with aripiprazole for schizophrenia patients and proves the latter has a significantly positive effect on the cognitive functions and psychological state of patients. In addition, these findings have important implications for clinical practice, as they suggest that a comprehensive treatment approach, incorporating both pharmacological and psychological interventions, may be more effective in managing schizophrenia. While our study highlights the short-term benefits of CBT combined with aripiprazole, future studies should consider a longer follow-up period (6–12 months) to better assess the sustained impact of this treatment approach. Additionally, future research could explore how continuous CBT or booster sessions might further enhance the durability of treatment effects. Such studies would provide valuable insights into the lasting benefits and optimal treatment strategies for schizophrenia. Furthermore, future research should investigate the potential mechanisms underlying the observed improvements in cognitive and psychological outcomes.

## Supporting information

Yan et al. supplementary material Yan et al. supplementary material

## Supplementary material

The supplementary material for this article can be found at https://doi.org/10.1017/neu.2025.14.
