# Supplementary material for: Application of cognitive behavioural therapy combined with aripiprazole in the treatment of schizophrenia: a randomised controlled trial
Source: Acta Neuropsychiatr. 2025 Mar 27;37:e57. doi: 10.1017/neu.2025.14 (PMC13130281; doi:10.1017/neu.2025.14)
Supplement: Yan et al. supplementary material [file S0924270825000146sup001.docx]

**Highlights**

1. CBT & aripiprazole alleviates the severity of symptoms in schizophrenia patients.
2. CBT & aripiprazole improves cognitive function in schizophrenia patients.

3. CBT & aripiprazole improves the mental state of schizophrenia patients.

4. CBT & aripiprazole improves the psychological state of schizophrenia patients.

5. CBT & aripiprazole improves the quality of life of schizophrenia patients.
